# Impact of Kefir Derived *Lactobacillus kefiri* on the Mucosal Immune Response and Gut Microbiota

**DOI:** 10.1155/2015/361604

**Published:** 2015-02-24

**Authors:** P. Carasi, S. M. Racedo, C. Jacquot, D. E. Romanin, M. A. Serradell, M. C. Urdaci

**Affiliations:** ^1^Université de Bordeaux, UMR 5248, Laboratoire de Microbiologie et Biochimie Appliquée (LBMA), Bordeaux Sciences Agro, 1 Cours du General de Gaulle, 33175 Gradignan, France; ^2^Cátedra de Microbiología, Departamento de Ciencias Biológicas, Facultad de Ciencias Exactas, Universidad Nacional de La Plata (UNLP), 47 y 115 s/n, 1900 La Plata, Argentina; ^3^Instituto de Estudios Inmunológicos y Fisiopatológicos (IIFP), CCT La Plata-CONICET, UNLP, 47 y 115 s/n, 1900 La Plata, Argentina

## Abstract

The evaluation of the impact of probiotics on host health could help to understand how they can be used in the prevention of diseases. On the basis of our previous studies and *in vitro* assays on PBMC and Caco-2 ccl20:luc reporter system presented in this work, the strain *Lactobacillus kefiri* CIDCA 8348 was selected and administrated to healthy Swiss mice daily for 21 days. The probiotic treatment increased IgA in feces and reduced expression of proinflammatory mediators in Peyer Patches and mesenteric lymph nodes, where it also increased IL-10. In ileum IL-10, CXCL-1 and mucin 6 genes were upregulated; meanwhile in colon mucin 4 was induced whereas IFN-*γ*, GM-CSF, and IL-1*β* genes were downregulated. Moreover, ileum and colon explants showed the anti-inflammatory effect of *L. kefiri* since the LPS-induced increment of IL-6 and GM-CSF levels in control mice was significantly attenuated in *L. kefiri* treated mice. Regarding fecal microbiota, DGGE profiles allowed differentiation of experimental groups in two separated clusters. Quantitative PCR analysis of different bacterial groups revealed only significant changes in *Lactobacillus* population. In conclusion, *L. kefiri* is a good candidate to be used in gut inflammatory disorders.

## 1. Introduction

Interactions between commensal bacteria, intestinal epithelial and immune cells play a crucial role in the maintenance of gut homeostasis [1, 2]. Microbial recognition through pattern-recognition receptors induces the expression and release of many different immune mediators, such as chemokines and pro- or anti-inflammatory cytokines which contribute to orchestrating both the innate and the adaptive immune response [3, 4]. The use of probiotics to modulate immune responses at mucosal and systemic level constitutes a very interesting alternative regarding the prevention and treatment of infectious diseases [5, 6] and different immunopathologies such as inflammatory bowel diseases and allergies [7–9] or metabolic disorders [10, 11].

Kefir grains are constituted by a complex symbiotic microbiota, and they are used to obtain fermented milks named “kefir” [12]. Several health-promoting properties such as immunological, antimicrobial, antitumoral, and hypocholesterolemic effects have been associated with kefir-consumption [13–17] and the study of the beneficial properties attributed to kefir-isolated microorganisms constitutes a field of great interest for the development of functional foods.

Immunomodulatory properties have been reported for different yeasts and bacteria isolated from kefir grains. Among kefir yeasts,* Kluyveromyces marxianus* CIDCA 8154 and* Saccharomyces cerevisiae* CIDCA 8112 downregulate intestinal epithelial innate response through a mechanism dependent on NF-kB modulation [18]. In the case of lactic acid bacteria retrieved from kefir,* L. kefiranofaciens* has been proven to ameliorate colitis in a DSS-induced murine model [19] and to produce antiasthmatic effects on ovalbumin-allergic asthma mice [20]. On the other hand, Carey and Kostrzynska [21] showed that* L. kefiri* attenuates the proinflammatory response in intestinal epithelial cells induced by* Salmonella* Typhimurium and Hong et al. [22] showed its influence on Th1 and proinflammatory cytokines production on macrophages.

One of the most important lactobacilli retrieved from kefir is* Lactobacillus kefiri* [23–26]. In previous studies, our workgroup has demonstrated that secretion products and surface proteins from* L. kefiri* exert a protective action against the invasion of* Salmonella enterica* serovar Enteritidis to Caco-2 cells [27] and also against the cytotoxic effects of clostridial toxins on Vero cells [28]. Moreover,* L. kefiri* strains have been proven to be safe [29] and to adhere to gastrointestinal mucus [30]. On the other hand,* L. kefiri* strains preserve a high percentage of viability after both spray-drying [31, 32] and freeze-drying procedures [33]. All the mentioned properties show the potentiality of* L. kefiri* as probiotic microorganism.

The study of the mechanisms underlying probiotic effect on the host on nonpathological conditions may be helpful for evaluating safety and further application of beneficial microorganisms in the prevention and treatment of different diseases. Taking into account the potentiality of* L. kefiri* as a novel probiotic, we propose to evaluate the immunomodulatory properties of kefir-isolated* L. kefiri* strains by* in vitro* and* in vivo* assays, along with changes in gut microbiota composition induced by* L. kefiri* administration.

## 2. Materials and Methods

### 2.1. Bacterial Strains and Growth Conditions

*Lactobacillus kefiri* CIDCA 83111, 83113, 83115, 8321, 8325, 8345, and 8348 were isolated from kefir grains [12].* L. kefiri* JCM 5818 was obtained from the Japanese Collection of Microorganisms (Reiken, Japan). Previously,* L. kefiri* CIDCA 83115, 8321, 8345, and 8348 were characterized as aggregating strains; meanwhile* L. kefiri* CIDCA 83111, 83113, and JCM 5818 were described as nonaggregative strains [34]. Lactobacilli were cultured in MRS-broth (DIFCO, Detroit, USA) 37°C for 48 h in aerobic conditions. Frozen stock cultures were stored at −80°C in skim milk until use.

### 2.2. Stimulation Assay with Caco-2 ccl20:luc Reporter System

The experiments were performed as described previously [35]. Briefly, Caco-2 cells stably transfected with a luciferase reporter construction under the control of CCL20 promoter (Caco-2 ccl20:luc) [36] were cocultured 2 h with a suspension of the* L. kefiri* strains (10^7^ CFU per well) to be tested (multiplicity of incubation = 100). Then, cells were stimulated using flagellin from* Salmonella enterica* ser. Typhimurium (FliC) (1 *μ*g mL^−1^) for 6 h. Luciferase activity was measured in a Labsystems Luminoskan TL Plus luminometer (Thermo Scientific, USA) using a luciferase assay system (Promega, Madison, WI, USA). Luminescence was normalized and expressed as the percentage of the mean of stimulated control (NAL).

### 2.3. PBMC Stimulation Experiments

Peripheral blood samples pretested for the absence of HIV or hepatitis virus infections were obtained from healthy volunteers (EFS Aquitaine, Bordeaux Blood Bank). Human PBMCs were isolated by centrifugation on Ficoll-Hypaque gradients. After washing, 2 × 10^6^ cells/well were cultured in 12-well plates in RPMI-1640 medium supplemented with 2 g L^−1^ NaHCO_3_, 300 mg L^−1^ L-glutamine, 100 *μ*g mL^−1^ streptomycin, 100 IU mL^−1^ penicillin (Sigma Chemical Co., St. Louis, MO, USA) and 10% FBS.

*L. kefiri* stimulation experiments on PBMC were performed coculturing 2 × 10^7^ bacteria per well (MOI = 10) during 24 h at 37°C in an atmosphere of 95% air and 5% CO_2_. Culture supernatants were collected and kept at −80°C until cytokines analysis. Experiences were realized in triplicate. Cell viability was not affected after 24 h of coincubation with bacteria (data not shown).

### 2.4. Quantification of Cytokine Levels in Culture Supernatants

Profiles of cytokines were analyzed after* L. kefiri* strain stimulation of PBMC using the Human Th1/Th2 11plex FlowCytomix Kit (eBioscience). It was designed to measure human IFN-*γ*, IL-1*β*, IL-2, IL-4, IL-5, IL-6, IL-8, IL-10, IL-12 p70, TNF-*α*, and TNF-*β*. Analysis was performed in a flow cytometer BD Accuri C6 (BD Biosciences). TGF-*β* was measured using the eBioscience human/mouse TGF beta 1 Ready-SET-Go! ELISA Kit (minimum detectable concentration 8.0 pg/mL).

### 2.5. Mice

Male Swiss albino mice, 4-week-old (Janvier, Le Genest St Isle, France), were quarantined 2 weeks after arrival and were housed under standard laboratory conditions with free access to food and water. The temperature was kept at 22°C and a 12-hour light/dark schedule was maintained. All procedures were performed according to the guidelines of the local ethics committee and in strict accordance with the guidelines issued by the European Economic Community “86/609.” Mice were randomly divided into two groups (*n* = 12/group) and received by gavage 10^8^ CFU of* L. kefiri* CIDCA 8348 (Lk group) or PBS (control group) daily for 7 days and 21 days; at each time point 6 mice of each group were sacrificed.

### 2.6. Tissue and Stool Sampling

Stools were collected at days 7, 14, and 21 and stored at −80°C until analysis. At the end of the experimental protocol, day 7 or 21, ileum and colon samples were collected and were preserved at −20°C in RNAlater (QIAGEN, Hilden, Germany) until RNA extraction. On day 21 Peyer Patches (PP) and mesenteric lymph nodes (mLN) were also removed and preserved at −20°C in RNAlater for expression analysis, and ileum and colon explants were collected in RPMI medium and processed immediately in order to analyze cytokines' secretion.

### 2.7. Quantification of Gene Expression in Tissue Samples by qRT-PCR

#### 2.7.1. RNA Extraction

Total RNA was isolated using the RNeasy Mini Kit (QIAGEN, Hilden, Germany) with an additional DNase treatment (Turbo DNA-free, Ambion, Inc.) according to the manufacturer's instructions.

#### 2.7.2. cDNA Synthesis

One *μ*g of total RNA was reverse-transcribed using the Maxima Reverse Transcriptase (Fermentas, France) with anchored-oligo (dT) 18 primer, according to manufacturers' instructions.

#### 2.7.3. Quantitative PCR

Quantitative real-time PCR analyses were performed using a CHROMO 4 System (Bio-Rad). The reaction mixture comprised Maxima SYBR Green/ROX qPCR Master Mix (Fermentas, France), 0.5 *μ*mol L^−1^ of each primer, and the respective standardized cDNA as a template. Target gene copy numbers were normalized against the housekeeping genes hypoxanthine phosphoribosyltransferase (HPRT) and *β*2 microglobulin (B2m). Cytokine and chemokine genes evaluated were* il1b, il6, il10, il12p70, il17a, il23, ifng, tnfa, tgfb, cxcl1, baff, april, gmcsf*; the transcription factors studied were* foxp3* and* rorgt; *epithelial barrier and IgA related genes were* zo-1, occludin, *and* pIgR*; mucin genes were* muc1, muc2, muc3, muc4, muc6, *and* muc13*. Primer sequences and PCR conditions are available upon request (E-mail: maria.urdaci@agro-bordeaux.fr). A negative control reaction without template was included for each primer combination.

### 2.8. Evaluation of Cytokine Secretion by Ileum and Colon Explants

Ileum and colon explants were cultured in RPMI medium supplemented with 10% fetal bovine serum (Gibco-Invitrogen, Carlsbad, CA, USA), 100 *μ*g mL^−1^ streptomycin and 100 IU mL^−1^ penicillin G, 100 *μ*g mL^−1^ gentamycin or RPMI complete medium with addition of 10 *μ*g mL^−1^ of LPS from* E. coli* as a stimulus (all from Sigma Chemical Co., St. Louis, MO, USA) for 24 h at 37°C in an atmosphere of 95% air and 5% CO_2_ [37]. Supernatants were collected, centrifuged, and frozen for later cytokines (IL-6, IL-4, IL-10, IL-17A, IFN-*γ*, and GM-CSF) measurements (Ready-SET-Go! ELISA Kit, eBioscience, France). All assays were performed according to the manufacturer's instructions. The minimum detectable concentrations were 4.0 pg mL^−1^ (IL-6, IL-4, and GM-CFS), 15 pg mL^−1^ (IFN-*γ*), and 30.0 pg mL (IL-10 and IL-17A).

### 2.9. Determination of Total IgA in Stools

At 7, 14, and 21 days after* L. kefiri* treatment the level of total IgA in stools was measured by ELISA according to the technique described by BD Pharmigen. Briefly, Maxisorp Nunc plates were coated overnight with purified rat anti-mouse IgA (BD 556969). The plates were washed with PBS containing 0.05% v/v Tween 20 (PBS-T) and blocked with FBS 10% v/v in PBS. Plates were incubated for 2 h at room temperature with purified mouse IgA kappa (BD 553476) or fecal samples. Plates were revealed using biotin rat anti-mouse IgA (BD 556978), streptavidin horseradish peroxidase (BD 554066), and trimethylbenzidine (TMB substrate reagent set BD OptEIA 555214). Using a Mutliscan FC microplate reader (Thermo Scientific) absorbance was read at 450 nm. All determinations were performed in triplicate.

### 2.10. Microbiota Population Analysis in Feces by q-PCR

Microbiota population analysis in feces was performed on the day 21 of the experience. DNA extraction was performed using the NucleoSpin Soil Genomic DNA isolation kit (Macherey-Nagel) according to the manufacturer's instructions except the feces solubilisation step. Quantification of bacterial populations was carried out using primers synthesized by Biomers (France). PCR reactions were performed on a CHROMO 4 System (Bio-Rad) using Maxima SYBR Green/ROX qPCR Master Mix (Fermentas, France). Twenty ng DNA and 0.2 *μ*mol L^−1^ of each primer were used in PCR mix. A negative control reaction without template was included for each primer combination. Melting curve was conducted from 70°C to 90°C read every 0.5°C during 2 s. The resulting data were collected and analyzed using Opticon Monitor. Standard curves were made with pure cultures of appropriate strains extracted using the same protocol as feces. Primers sequences are able on Table 1.

### 2.11. Qualitative Analysis of Fecal Microbiota by PCR-DGGE

HDA1 and HDA2-GC (GC clamp required for DGGE analysis [38], targeting the V2-V3 region [39]) were used to assess microbial diversity in each sample. The PCR products were separated in 8% polyacrylamide gels (37.5 : 1 acrylamide : bisacrylamide) with a range of 30–50% denaturing gradient (100% denaturant consisted of 7 M urea and 40% deionized formamide) cast with Bio-Rad's Model 475 gradient delivery system (BioRad, Hercules, CA, USA). The electrophoresis was performed in TAE 0.5X buffer for 5 h at a constant electric current of 125 mA and a temperature of 60°C with the DCode Mutation Detection System (Bio-Rad, Hercules, CA, USA). Clustering analysis was performed using the UPGMA (unweighted pair group method with arithmetic mean clustering algorithm) to calculate the dendrograms.

### 2.12. Statistical Analysis

Statistical comparisons for significant differences were performed according to Student's *t*-test. Differences with *P* < 0.05 were considered significant.

## 3. Results

### 3.1. Cytokines Profile of PBMC Cocultured with* L. kefiri* Strains

A preliminary screening of the eight* L. kefiri* strains was carried out using PMBC. PBMC and bacteria coculture assays were performed and profiles of cytokines secreted during incubation with the strains were analyzed. The levels of IL-2, IL-4, IL-5, TNF-*β* y TGF-*β*1 were under the lower range of reliable detection. Meanwhile a significant increase in IL-1*β*, IL-6, IL-10, TNF-*α*, IL-8, and IL-12 p70 concentrations was observed for all tested microorganisms (Table 2). In an attempt to predict the type of Th response they could promote, we analyzed the TNF-*α*/IL-10 and IL-10/IL-12 ratios (Table 3).

The highest TNF-*α*/IL-10 ratio was observed for the nonaggregating strain* L. kefiri* JCM 5818 and the lowest for the autoaggregative strain* L. kefiri* CIDCA 8348. In agreement with these results,* L. kefiri* CIDCA 8348 showed the highest IL-10/IL-12 ratio while* L. kefiri* JCM 5818 was, among other strains such as CIDCA 83111, 83113, and 83115, in the opposite ratio, expecting a poor anti-inflammatory effect.

### 3.2. Regulation of Caco-2 ccl20:luc Reporter System by* L. kefiri* Strains

**Figure 1:**
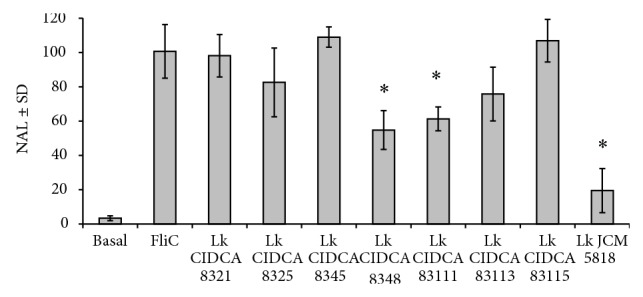
Modulation of proinflammatory response in Caco-2 ccl20:luc reporter system by* L. kefiri* strains. NAL: normalized average luminescence expressed as percentage of activity induced with flagellin stimulation; FliC:* Salmonella*-isolated flagellin; Basal: without any stimulation. Results are expressed as mean ± standard deviation and are representative of at least three independent experiments. ^*^ *P* < 0.01.

The ability of the eight strains of* L. kefiri* to modulate intestinal innate response to proinflammatory stimuli such as flagellin (FliC) was studied using a Caco-2 ccl20:luc reporter system [18, 36]. Only three strains (CIDCA 8348, 83111, and JCM 5818) downregulated cell activation induced by FliC (Figure 1), suggesting their potential anti-inflammatory properties.

*L. kefiri* CIDCA 8348 was chosen to perform* in vivo* studies on Swiss mice since parameters associated with safety and other beneficial properties have been previously demonstrated [29]. Moreover,* L. kefiri* CIDCA 8348 is an aggregative strain. This is an important property for probiotics since it has been proposed that aggregation represents a mechanism by which gastrointestinal commensals adhere to each other and it could allow them to colonize persistently in biofilms on the host's mucosa [40].

### 3.3. Kinetics of Fecal IgA Response after Oral Administration of* L. kefiri* CIDCA 8348 in Swiss Mice

**Figure 2:**
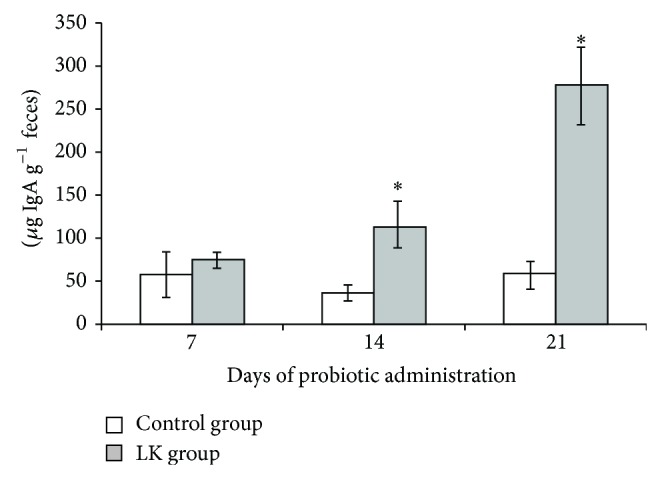
IgA quantification from fecal samples taken on day 7, 14, or 21 from control mice and* L. kefiri* treated mice (Lk). Results are expressed as mean ± standard deviation. ^*^ *P* < 0.05.

Stool suspensions were assayed for total IgA by ELISA to evaluate the induction of mucosal IgA (Figure 2). An induction was observed after 14 days of probiotic administration and the levels continue rising after 21 days. Even though no differences in IgA secretion were observed after 7 days of treatment between groups, flow cytometry quantified IgA^+^ cells were significantly higher in mLN from Lk group (data not shown).

### 3.4. Effect of* L. kefiri* Administration on Gene Expression of Gut Mucosa

The expression of cytokines, chemokines, mucins, and epithelial barrier genes as well as IgA related genes was studied by qRT-PCR in ileum and colon after 7 and 21 days of oral administration of* L. kefiri* CIDCA 8348.

**Figure 3:**
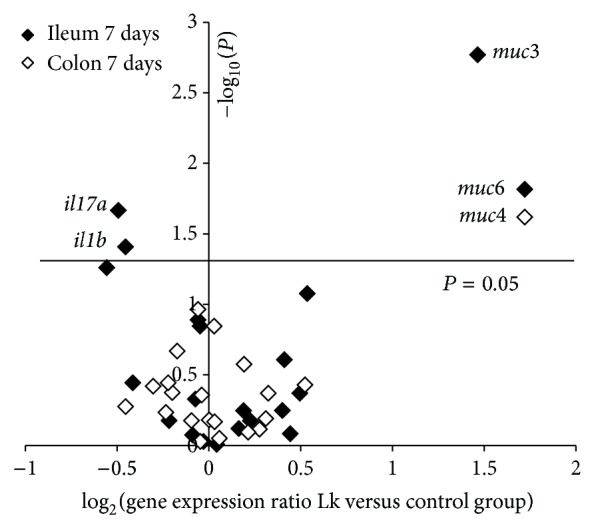
Gene expression ratio in ileum (black) and colon (white) of Lk group* versus* control group after 7 days of* L. kefiri* administration. The* x*-axis of the plot represents log_2_ relative expression level of the gene and the* y*-axis displays the −log_10_ *P* (statistical significance). The names of the genes which displayed significant differences are included.

As shown in Figure 3, a seven-day treatment significantly downregulated IL-1*β* and IL-17A gene expression in ileum; meanwhile mucin 3 and mucin 6 were upregulated. In contrast, in colon only gene expression of mucin 4 was modified.

**Figure 4:**
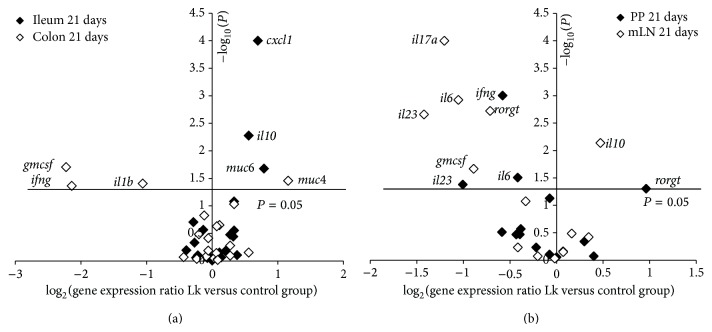
Gene expression ratio of Lk group* versus* control group after 21 days of* L. kefiri* administration. The* x*-axis of the plot represents log_2_ relative expression level of the gene and the* x*-axis displays the −log_10_ *P* (statistical significance). The names of the genes which displayed significant differences are included. (a) Expression in ileum (black) and colon (white). (b) Expression in PP (black) and mLN (white).

The administration of* L. kefiri* for a longer period, 21 days, produced higher expression levels of IL-10, CXCL-1, and mucin 6 genes in ileum (Figure 4(a)). In colon, downregulation of IFN-*γ*, GM-CSF, and IL-1*β* genes was observed together with the upregulation of mucin 4 (Figure 4(a)).

The effect of* L. kefiri* treatment for 21 days on gene expression was also evaluated in Peyer patches (PP) and mesenteric lymph nodes (mLN) (Figure 4(b)). In PP the expression of IL-23, IFN-*γ*, and IL-6 was downregulated. Interestingly, in mLN not only proinflammatory mediators (IL-6, IL-23, IL-17A, and GM-CSF) and ROR*γ*t transcription factor were downregulated but also IL-10 gene expression was increased.

### 3.5. *Ex Vivo* Mice Intestinal Explants to Study Mucosal Anti-Inflammatory Effect of* L. kefiri*

**Figure 5:**
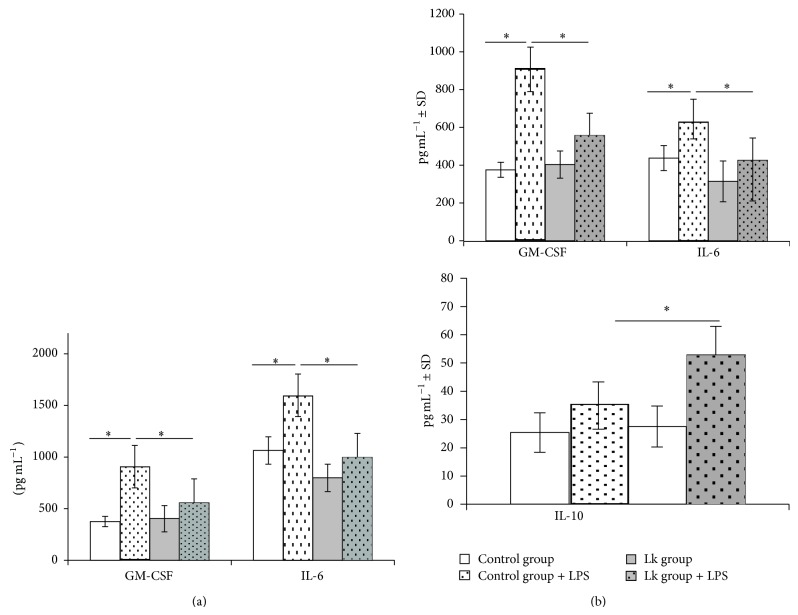
Cytokine's release in supernatants of (a) ileum and (b) colonic explants cultured for 24 h in the presence of LPS. Results are expressed as mean ± standard deviation. ^*^ *P* < 0.05.

To analyze the ability of* L. kefiri* treatment to modulate the mucosal immune response in a proinflammatory environment,* ex vivo* experiments were performed stimulating ileum and colon explants with LPS from not treated (control) and 21-day* L. kefiri* treated mice. LPS stimulation induced an increment of IL-6 and GM-CSF levels in control mice (Figures 5(a) and 5(b)). These increments were significantly attenuated in both ileum and colon explants of* L. kefiri* treated mice (Figures 5(a) and 5(b)). Moreover, in colon explants from Lk group a higher secretion of IL-10 was observed in LPS stimulated samples (Figure 5(b)). The levels of IL-4, IL-17, IFN-*γ*, and TNF-*α* were undetectable in both Lk and control mice explants.

### 3.6. Effect of* L. kefiri* Administration on Fecal Microbiota

**Figure 6:**
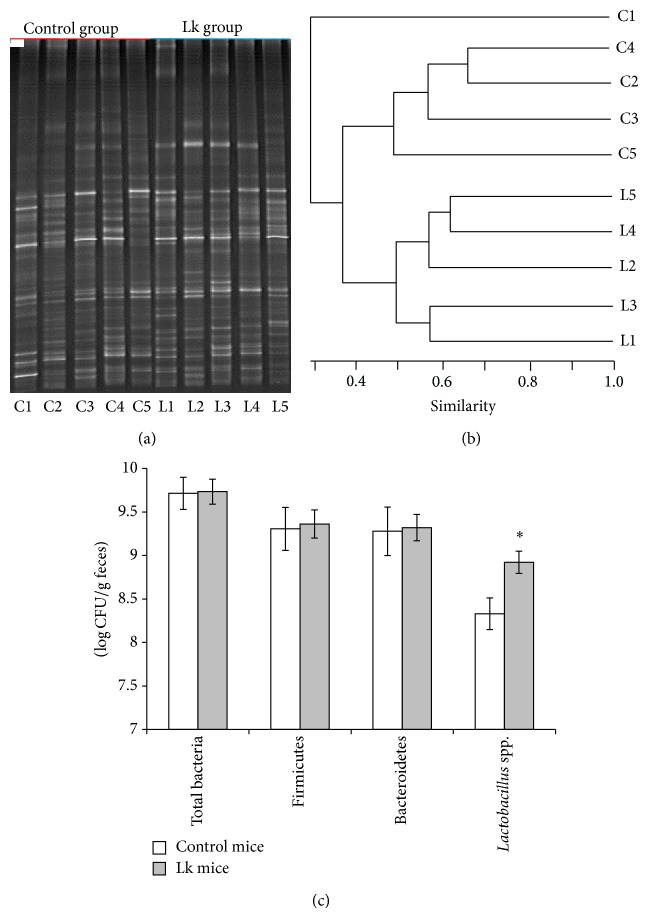
Evaluation of microbiota on fecal samples taken on the 21st day of trial from control and Lk groups. (a) Total bacteria DGGE profiles of five mice from control group (lanes C1 to C5) and five from Lk group (lanes L1 to L5). (b) Dendrogram for the total bacterial DGGE profiles. Clustering analysis was performed using the UPGMA linkage. (c) qPCR quantification of total bacteria,* Firmicutes, Bacteroidetes,* and* Lactobacillus* spp. Results are expressed as mean ± standard deviation. ^*^ *P* < 0.05.

The qualitative profile of fecal microbiota was determined by PCR-DGGE (Figure 6(a)). Microbial diversity was assessed by the number of amplification bands generated from each sample. There were no differences between control and Lk group (32 ± 3 and 30 ± 2, resp.). However, changes in the microbial community composition were produced since the cluster analysis based on the Pearson product-moment correlation coefficient and UPGMA linkage allowed differentiation of the experimental groups in two clusters (Figure 6(b)).

As expected, an increment in* Lactobacillus* population was observed by qPCR but quantitative differences were not observed in the two major phyla, Firmicutes or Bacteroidetes (Figure 6(c)). Moreover, no significant changes were detected in other evaluated bacterial populations (Table 1).

## 4. Discussion

In the last years, an increasing number of* in vitro* and* in vivo* experiments have supported the idea that probiotic microorganisms confer their health benefits to the host by interacting with the immune system, particularly through establishing and maintaining a balance between pro- and anti-inflammatory cytokines [41, 42]. In kefir, bacteria and yeasts exist in symbiotic association and contributed to beneficial properties. Several authors have demonstrated the ability of kefir to modulate the mucosal immune response in mice and suggest that a Th1 response was controlled by Th2 cytokines [15, 16]. Some immunological effects were attributed to the formation of bioactive peptides during milk fermentation and also to production of exopolysaccharides as kefiran [13]. However, features regarding the effects of bacteria remain very important. It has been recently described that one strain of* L. kefiranofaciens* protects mice in a model of allergy [20] and also in an experimental model of colitis [19], but to our knowledge, our work constitutes the first report of the* in vivo* immunomodulatory activity of* L. kefiri*.

In the present work we demonstrated that* L. kefiri* strains induced the secretion of proinflammatory Th1 mediators such as IL-1*β*, IFN-*γ*, IL-6, IL-12p70, and TNF-*α* in PBMC as well as the production of the Th2 cytokine IL-10. These findings are not surprising, since several authors have reported the upregulation of these proinflammatory cytokines by probiotic bacteria on PBMC [6, 43–45] or in mice macrophages by* L. kefiranofaciens* [22]. However, we found that* L. kefiri* strains stimulate immune cells to produce different ratios of cytokines, suggesting that they could possess different T cell polarizing abilities.

Cytokines are mutually regulated molecules; thus the balance between them influences CD4+ T-cell differentiation towards Th1, Th2, or Th17 cells. IL-12 induces Th1-mediated responses; meanwhile the anti-inflammatory cytokine IL-10 suppresses the production of IL-12 among other Th1 cytokines. The observed differences in the production of IL-12, IL-10, and TNF-*α* could contribute to understanding the type of response a strain may promote [45, 46]. JCM 5818 showed the highest TNF-*α*/IL-10 ratio whereas CIDCA 8348 presented the lower ratio. Moreover, CIDCA 8348 showed also the highest IL-10/IL-12 ratio which presupposes that it is a good anti-inflammatory candidate [47]. In concordance with these results, the strain CIDCA 8348 was also capable, along with other two* L. kefiri* strains, of eliciting an anti-inflammatory response on flagellin-stimulated intestinal epithelial cells (Caco-ccl20 reporter system) which has been previously reported for several probiotic bacteria [48] and yeasts [18, 49]. Curiously, JCM 5818 strain that presented the most anti-inflammatory capacity using Caco-ccl20 reporter system presented the most proinflammatory profile using PBMC. It might be interesting in the future to study the* in vivo* anti-inflammatory properties of this strain.

Although* in vitro* research using PBMC from healthy donors or intestinal epithelial cells can be used to screen the immunomodulatory activity of probiotic strains candidates, while reducing considerably the use of animals for screening purposes, they could not always be a good indicator of* in vivo* effect [4, 46, 47]. In consequence, to better understand the immunomodulatory ability of* L. kefiri*, the strain CIDCA 8348 was selected to be administered orally to mice in order to analyze the effect on different aspects of mucosal immune response and microbiota modulation.

CIDCA 8348 strain occasioned an increment in IgA+ B cells in mLN and it correlated with an increase of IgA in fecal samples of* L. kefiri*-treated mice. These findings are in agreement with results reported for some lactobacilli-based probiotics [50, 51] or even for the administration of kefir-fermented milk [16, 52]. SIgA, the predominant immunoglobulin in secretions, is a key element in maintaining gut homeostasis and in the protection of mucosal surfaces against pathogens [53]. Expression of molecules involved in class switch to IgA, expansion of IgA-expressing B cells, and their differentiation to IgA secreting plasma cells was studied. Even though no changes in the expression of APRIL, BAFF, and TGF*β*1 genes in PP, mLN, ileum, or colon were observed, IL-10 was significantly induced in both ileum and mLN. It has been described that this cytokine induces IgA production, either through induction of TGF*β* within the target B cell itself or through enhancement of the postswitch maturation [54]. Nevertheless, a downregulation of the expression of proinflammatory cytokines (IL-1*β* and IL-17A) was observed in ileum tissue at 7th day of administration of* L. kefiri*. This effect became more evident after 21 days of treatment, when a significant decrease of several proinflammatory mediators was determined in Peyer's patches (IL-6, GM-CSF, IL-17A, and IFN-*γ*), mesenteric lymphoid nodes (IL-6, GM-CSF, and IL-17A), and colon (GM-CSF, IFN-*γ*, and IL-1*β*) showing the anti-inflammatory ability of this* L. kefiri* strain* in vivo*. This kind of results, which support the suppression of proinflammatory immunity by probiotics, was reported for different nonpathogenic and probiotic bacteria by other authors in healthy [55] or disease models [47], but this is the first report for* L. kefiri* isolated from kefir. Moreover, the anti-inflammatory cytokine IL-10 was increased in ileum as well as the chemokine CXCL-1. This interesting chemoattractant, analogous in function to human IL-8, is an important regulator of neutrophil recruitment from the lamina propria to the epithelium and has been shown to be essential in protection against DSS-induced colitis [56].

On the other hand, intestinal explants from* L. kefiri*-treated mice showed a downregulation of IL-6 and GM-CSF after* in vitro* stimulation with a proinflammatory mediator such as LPS in comparison with control mice. Taken together, all these experiments allowed us to confirm the anti-inflammatory phenotype associated with* L. kefiri* CIDCA 8348 administration.

Regarding another feature on mucosal physiology, we studied the effect of* L. kefiri* administration on the expression of mucin genes. Mucins are the main component of the mucus layer and it has been described that their secretion could be modified by changes in host microbiota, infections, and probiotic or antibiotic treatments [57–59]. Only a few authors have evaluated the effect of probiotic administration in healthy lab animals. Particularly, Dykstra et al. [60] observed differential induction of* muc1, muc2,* and* muc3* in ileum and colon after administration of* Lactobacillus plantarum* 299 v to Sprague-Dawley rats. In addition, studies performed in Swiss mice revealed that administration of* L. plantarum* L91 induced* muc2* in colon [61]; meanwhile Jiang et al. [62] reported that* L. rhamnosus* GG-treated C57BL/6NHsd mice overexpressed* muc3* without changes in m*uc1, muc2,* or* muc4*. In* L. kefiri*-treated mice* muc3* and* muc6* increased their expression in the ileum after 7 days of treatment whereas at 21 days only* muc6* was increased. In colon, at 7 and 21 days* muc4* expression was increased in* L. kefiri*-treated mice. These changes could be associated with the presence of* L. kefiri* in the gut or with the modifications in microbiota populations induced by it [63]. Moreover, differences in the quantity and composition of the local microbiota [64] as well as the characteristics and thickness of the mucus layer [58, 65] could have an impact in the way* L. kefiri* interacts with the epithelium or its effect on microbiota.

## 5. Conclusion

In this study, we demonstrated that* L. kefiri* strains isolated from kefir stimulated the production of different ratios of pro/anti-inflammatory cytokines* in vitro*. We proved that the administration of* L. kefiri* CIDCA 8348 to mice not only downregulates expression of proinflammatory mediators but also increases anti-inflammatory molecules in gut immune system inductive and effector sites. Likewise, the increment in IgA production together with mucin induction and the impact in microbiota demonstrate the importance of this probiotic in the regulation of intestinal homeostasis. Thus, it is a good candidate to be used in gut inflammatory disorders.

## Figures and Tables

**Table 1:** Sequences of oligonucleotide primers.

Population	Forward and reverse primers (5′-3′)	Reference
Total bacteria (HDA)	ACTCCTACGGGAGGCAGCAG GTATTACCGCGGCTGCTGGCAC	[39]
*Lactobacillus* group	AGCAGTAGGGAATCTTCCA ATTYCACCGCTACACATG	

*Firmicutes *	GGAGYATGTGGTTTAATTCGAAGCA AGCTGACGACAACCATGCAC	[66]
*Bacteroidetes *	GGARCATGTGGTTTAATTCGATGAT AGCTGACGACAACCATGCAG	

*Faecalibacterium prausnitzii *	AGATGGCCTCGCGTCCGA CCGAAGACCTTCTTCCTCC	[67]

*Escherichia coli *	CATGCCGCGTGTATGAAGAA CGGGTAACGTCAATGAGCAAA	[68]

*Prevotella* group	CACCAAGGCGACGATCA GGATAACGCCYGGACCT	[69]

*Clostridium leptum* group	GCACAAGCAGTGGAGT CTTCCTCCGTTTTGTCAA	[70]

*Enterococcus* spp.	CCCTTATTGTTAGTTGCCATCATT ACTCGTTGTACTTCCCATTGT	
*Clostridium coccoides* group	CGGTACCTGACTAAGAAGC CTTCCTCCGTTTTGTCAA	[71]
*Bifidobacterium* spp.	TCGCGTCYGGTGTGAAAG CCACATCCAGCRTCCAC	

*Bacteroides fragilis* group	CTGAACCAGCCAAGTAGCG CCGCAAACTTTCACAACTGACTTA	[72]

*Segmented filamentous bacteria *	GACGCTGAGGCATGAGAGCAT GACGGCACGGATTGTTATTCA	[73]

*Lactobacillus murinus *	GTGGCGAACGGGTGAGTAA GCACCTGTTTCCAAGTGTTATCC	[74]

*Akkermansia muciniphila *	CAGCACGTGAAGGTGGGGAC CCTTGCGGTTGGCTTCAGAT	[75]

**Table 2:** Cytokine production after exposing PMBCs for 24 h to *L. kefiri* strains. Cytokines concentrations in culture cell supernatant (pg mL^−1^) were measured using Flow Human Th1/Th2 11plex FlowCytomix Kit (eBioscience). The results are expressed as mean ± SD of experiments performed with three different donors.

*L. kefiri *	IL-1*β*	IL-6	IL-8	IL-10	IFN-*γ*	TNF-*α*	IL-12p70
CIDCA 8321	1294 ± 526	1552 ± 709	5771 ± 1284	205 ± 76	131 ± 22	10436 ± 3785	312 ± 72
CIDCA 8325	2050 ± 75	2571 ± 94	4824 ± 531	313 ± 11	59 ± 38	16169 ± 45	572 ± 94
CIDCA 8345	1655 ± 8	2033 ± 15	4399 ± 106	230 ± 3	85 ± 20	15368 ± 1075	449 ± 21
CIDCA 8348	1936 ± 10	2719 ± 13	3855 ± 40	435 ± 90	83 ± 4	13551 ± 198	502 ± 121
CIDCA 83115	1023 ± 60	1778 ± 12	3621 ± 34	192 ± 9	49 ± 2	8613 ± 500	738 ± 206
CIDCA 83111	604 ± 83	2401 ± 81	3806 ± 167	253 ± 1	103 ± 23	9908 ± 175	815 ± 189
CIDCA 83113	1148 ± 26	1722 ± 95	3920 ± 202	201 ± 11	53 ± 22	7514 ± 427	475 ± 59
JCM 5818	591 ± 103	919 ± 40	4228 ± 12	84 ± 2	62 ± 13	6872 ± 1647	246 ± 94
Nonstimulated PBMC	35 ± 2	71 ± 6	418 ± 202	21 ± 1	15 ± 11	175 ± 5	41 ± 13

**Table 3:** TNF-*α*/IL-10 and IL-10/IL-12 ratio determined after *in vitro* PBMC stimulation with *L. kefiri* strains. Means with the same letter for each parameter are not significantly different.

*L. kefiri *	TNF-*α*/IL-10	IL-10/IL-12
CIDCA 8321	50.9 ± 11.4^c,d^	0.66 ± 0.24^d,e,f^
CIDCA 8325	51.7 ± 0.1^d^	0.55 ± 0.06^e^
CIDCA 8345	66.8 ± 4.7^e^	0.51 ± 0.02^e^
CIDCA 8348	31.2 ± 0.5^b^	0.87 ± 0.18^f^
CIDCA 83115	44.9 ± 2.6^c^	0.26 ± 0.01^b^
CIDCA 83111	39.2 ± 0.7^c^	0.31 ± 0.02^c^
CIDCA 83113	37.4 ± 2.1^c^	0.42 ± 0.02^d^
JCM 5818	81.8 ± 19.6^f^	0.34 ± 0.03^c^
Nonstimulated PBMC	8.3 ± 0.2^a^	0.005 ± 0.002^a^

## Abbreviations

PBMC: Peripheral blood mononuclear cells
GM-CSF: Granulocyte macrophage colony-stimulating factor
CXCL-1: Chemokine (C-X-C motif) ligand 1
IL: Interleukin
IFN: Interferon
TNF: Tumor Necrosis Factor.
